# Case Report: Pathologic Complete Response to Induction Therapy in a Patient With Potentially Resectable Pancreatic Cancer

**DOI:** 10.3389/fonc.2022.898119

**Published:** 2022-06-06

**Authors:** Changchang Lu, Yahui Zhu, Hao Cheng, Weiwei Kong, Linxi Zhu, Lei Wang, Min Tang, Jun Chen, Qi Li, Jian He, Aimei Li, Xin Qiu, Dongsheng Chen, Fanyan Meng, Xiaoping Qian, Baorui Liu, Yudong Qiu, Juan Du

**Affiliations:** ^1^ The Comprehensive Cancer Center of Drum Tower Hospital, Medical School of Nanjing University, Nanjing, China; ^2^ Nanjing Drum Tower Hospital Clinical College of Nanjing University of Chinese Medicine, Nanjing, China; ^3^ Department of Hepatopancreatobiliary Surgery, Drum Tower Hospital, Medical School of Nanjing University, Nanjing, China; ^4^ Digestive Department of Drum Tower Hospital, Medical School of Nanjing University, Nanjing, China; ^5^ Imaging Department of Drum Tower Hospital, Medical School of Nanjing University, Nanjing, China; ^6^ Pathology Department of Drum Tower Hospital, Medical School of Nanjing University, Nanjing, China; ^7^ Nuclear Medicine Department of Drum Tower Hospital, Medical School of Nanjing University, Nanjing, China; ^8^ The State Key Laboratory of Translational Medicine and Innovative Drug Development, Medical Department, Jiangsu Simcere Diagnostics Co., Ltd., Nanjing, China

**Keywords:** potentially resectable pancreatic cancer, induction therapy, PD-1 inhibitor, pathologic complete response, benefit

## Abstract

Immune monotherapy does not appear to work in patients with pancreatic cancer so far. We are conducting a clinical trial that combines programmed cell death protein-1 (PD-1) inhibitor with chemotherapy and concurrent radiotherapy as induction therapy for patients with locally advanced pancreatic cancer (LAPC) and borderline resectable pancreatic cancer (BRPC). Here, we report a case with a pathologic complete response (pCR) and no postoperative complications after the induction therapy. The patient received four cycles of induction therapy and achieved a partial response (PR) with a significant decline of tumor marker carbohydrate antigen 19-9 (CA19-9). Also, peripheral blood samples were collected during the treatment to investigate serial circulating tumor DNA (ctDNA) dynamic changes in predicting the tumor response and outcomes in patients. Our result suggested that PD-1 blockade plus chemotherapy and concurrent radiotherapy is a promising mode as induction therapy for patients with potentially resectable pancreatic cancer. In this case, serial ctDNA alterations accurately provide a comprehensive outlook of the tumor status and monitor the response to the therapy, as validated by standard imaging.

## Introduction

Pancreatic cancer is one of the worst poor-prognosis gastrointestinal tumors with the lowest 5-year relative survival rate (10%) (1). According to the cancer incidence and deaths in the United States, pancreatic cancer is projected to become the second leading cause of cancer-related death by 2030 (2). So far, surgery remains the first option for patients with resectable pancreatic cancer, while only less than 20% of the patients may be possible to have surgery at definite diagnosis (3). Chemoradiation is still recommended as the best choice for locally advanced and metastatic disease (4). The National Comprehensive Cancer Network (NCCN) guidelines have endorsed neoadjuvant therapy for patients with resectable or borderline resectable pancreatic cancer (BRPC).

Recently, immunotherapy especially the immune checkpoint inhibitors has been a research hot spot (5). There is research suggesting that first-line pembrolizumab monotherapy significantly improved the overall survival (OS) and progression-free survival in patients with metastatic non-small-cell lung cancer (NSCLC) (6). In addition, a previous study has found that pancreatic cancer treated with chemotherapy plus immunotherapy has a higher survival rate in comparison to chemotherapy alone based on the exploration of the signaling pathways involved in Pancreatic Ductal Adenocarcinoma(PDAC) development (7). Moreover, other types of immunotherapies including tumor vaccine (8) and chimeric antigen receptor T-cell (CAR-T) therapy (9) have also gradually emerged in antitumor therapy. However, all kinds of immunotherapies failed to make a breakthrough in the treatment of pancreatic cancer because of its unique immunosuppressive tumor microenvironment (TME) (10). There are some findings that indicate that radiation may induce antitumor immunity (11), promote the recruitment of effective T cells, and enhance the susceptibility of tumor cells to T cell-mediated antitumor effects (12). The combination of radiation and immune checkpoint blockades has increased T-cell priming and augmented activation in a poorly immunogenic mouse model of pancreatic cancer (13).

Advances in neoadjuvant therapy and induction therapy in various tumors open a new avenue to improve the resection rate of pancreatic cancer and prolong survival. A growing body of study shows that patients with BRPC or locally advanced pancreatic cancer (LAPC) tend to benefit from neoadjuvant or induction therapy, which increases the rate of margin-negative resections and decreases recurrence rates (14–16). Based on preliminary research and the curative effects in clinical patients treated with different therapeutic schedules, we conducted a clinical trial on the combination of PD-1 blockade and concurrent radiotherapy for patients with potentially resectable pancreatic cancer, mainly referring to BRPC and LAPC.

Circulating tumor DNA (ctDNA) has been proven to have worth in multiple perspectives in pancreatic cancer (17). For resected pancreatic cancers, postoperative ctDNA is applied in predicting prognosis (18). As for localized and advanced pancreatic cancers, the value of ctDNA is monitoring relapse or progression (19). On account of the noninvasive advantages, ctDNA could provide longitudinal and dynamic surveillance of the tumor-specific genetic characteristics. In this case, serial ctDNA samples were collected to identify genetic alterations in baseline and explore the predictive role of ctDNA in pancreatic cancer patients treated with immunological induction therapy.

Here, we report a case with a clear beneficial outcome from this treatment that exhibited a pathologic complete response (pCR) and a significant decrease in tumor index. Serial ctDNA alterations accurately provide a comprehensive outlook of the tumor status and monitor the response to the therapy.

## Case Presentation

A 62-year-old man was hospitalized for surgery for intermittent epigastric pain in September 2019. Tumor markers carbohydrate antigen 19-9 (CA19-9) and carbohydrate antigen 125 (CA125) were 3,958 U/ml and 50.3 U/ml, respectively. Contrast-enhanced computed tomography (CT) showed a mass at the head of the pancreas, this mass was suspected to have invaded the superior mesenteric vein, but no distant metastasis was detected. Positron emission tomography computed tomography (PET-CT) further exhibited a mass and hypermetabolism of glucose in the uncinate process of the pancreas (Size = 4.1 cm * 3.6 cm; SUV = 18.2) and has an unclear boundary with the duodenum. The result of an endoscopic ultrasound-guided fine-needle aspiration biopsy (EUS-FNA) was concordant with adenocarcinoma, and the patient was diagnosed with potentially resectable pancreatic cancer. After discussion, the Multiple Disciplinary Team (MDT) recommended him to participate in our clinical trial of induction therapy.

**Figure 1:**
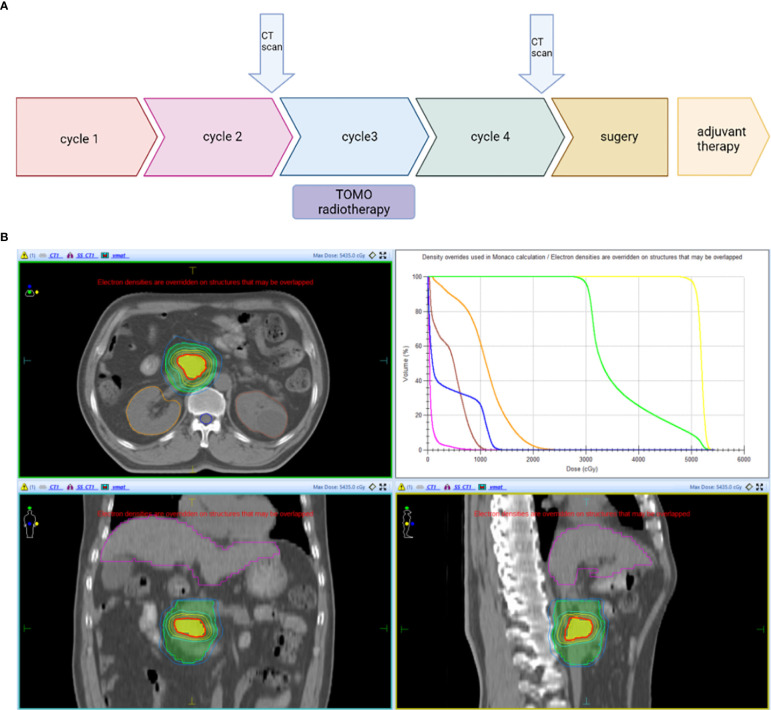
**(A)** Timeline of the treatment: Every 3 weeks one cycle: Tislelizumab (200 mg, on day 1), gemcitabine (1,000 mg/m^2^, on days 1 and 8), and nab-paclitaxel (125 mg/m^2^, on days 1 and 8). **(B)** Radiotherapy: PTV: 3 Gy * 10f; PGTV: 5 Gy * 10f. CT scan, Computed Tomography scan; PTV, planning target volume; PGTV, planning gross tumor volume.

**Figure 2:**
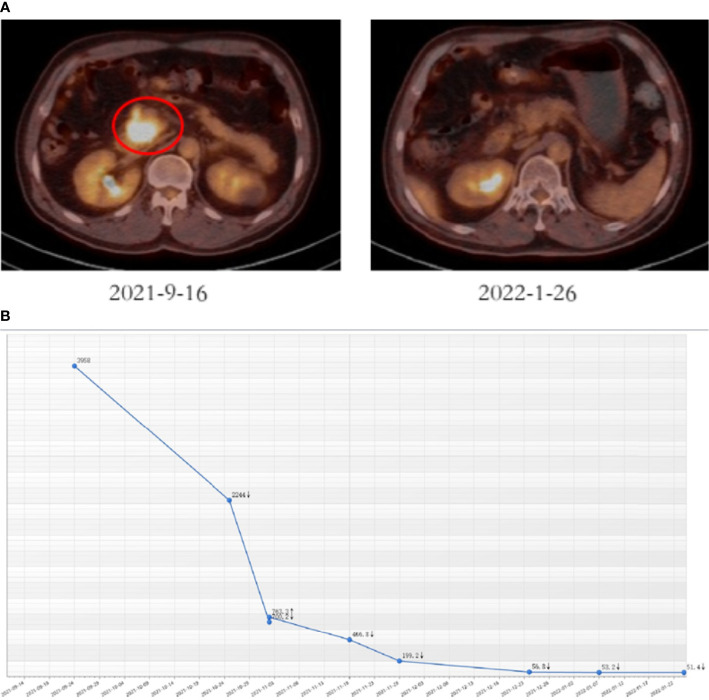
Tumor responses after induction therapy. **(A)** PET-CT was performed before (size: 4.1 cm * 3.6 cm, SUV = 18.2) and after 4 cycles of the treatment (size: 2.3 cm * 1.5 cm, SUV = 4.8). **(B)** CA19-9 levels of this patient during the treatment.

**Figure 3:**
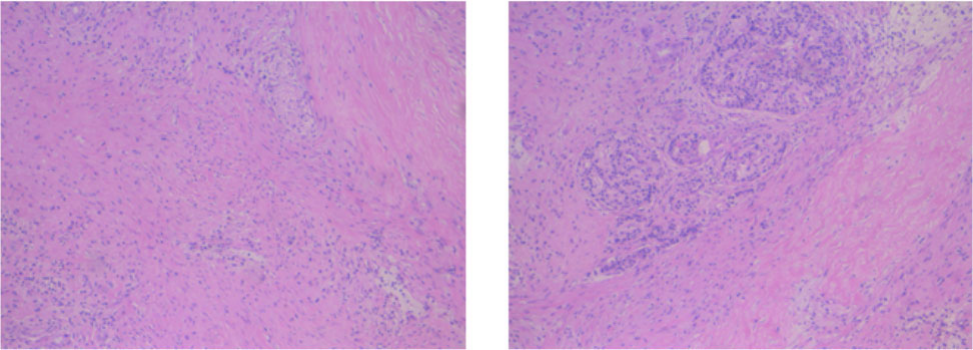
Postoperative pathology of the patient. No residual tumor cells, and the surgical margin was negative.

The patient firstly received 4 cycles of gemcitabine 1,000 mg/m^2^ and nab-paclitaxel 125 mg/m^2^ on day 1 and day 8, along with tislelizumab (a PD-1 monoclonal antibody) 200 mg on day 1 every 3 weeks, concurrently with tomotherapy (TOMO) with a total radiation dose of 30 Gy/10f at the planning target volume (PTV) and 50 Gy/10f at the planning gross tumor volume (PGTV) during the third cycle. Timeline of events and the radiation target volume and precise radiotherapy planning are detailed in **Figure 1**. The patient had repeat CT about every two cycles. After 4 cycles of the treatment, PET-CT revealed that the pancreatic tumor size and glucose metabolism had decreased remarkably, and glucose metabolism in peripheral lymph nodes returned to normal **(** **Figure 2** **A** **)** . As shown in **Figure 2B**, tumor marker CA19-9 decreased to 51.4 U/ml. The curative effect was evaluated as partial response (PR). Based on these signs of improvement, the MDT agreed to give him surgical exploration. Surprisingly, not only did the patient receive pylorus-preserving pancreaticoduodenostomy (PPPD) successfully but also the postoperative pathology showed a pCR **(** **Figure 3** **)**. Neither residual tumor cells nor metastases in 16 regional lymph nodes were detected, and the surgical margin was negative. The final TNM stage was ypT0N0M0. Better yet, the patient had no serious postoperative complications and recovered smoothly. We planned to give him at least 2 cycles of adjuvant therapy at postoperative 1 month.

**Figure 4:**
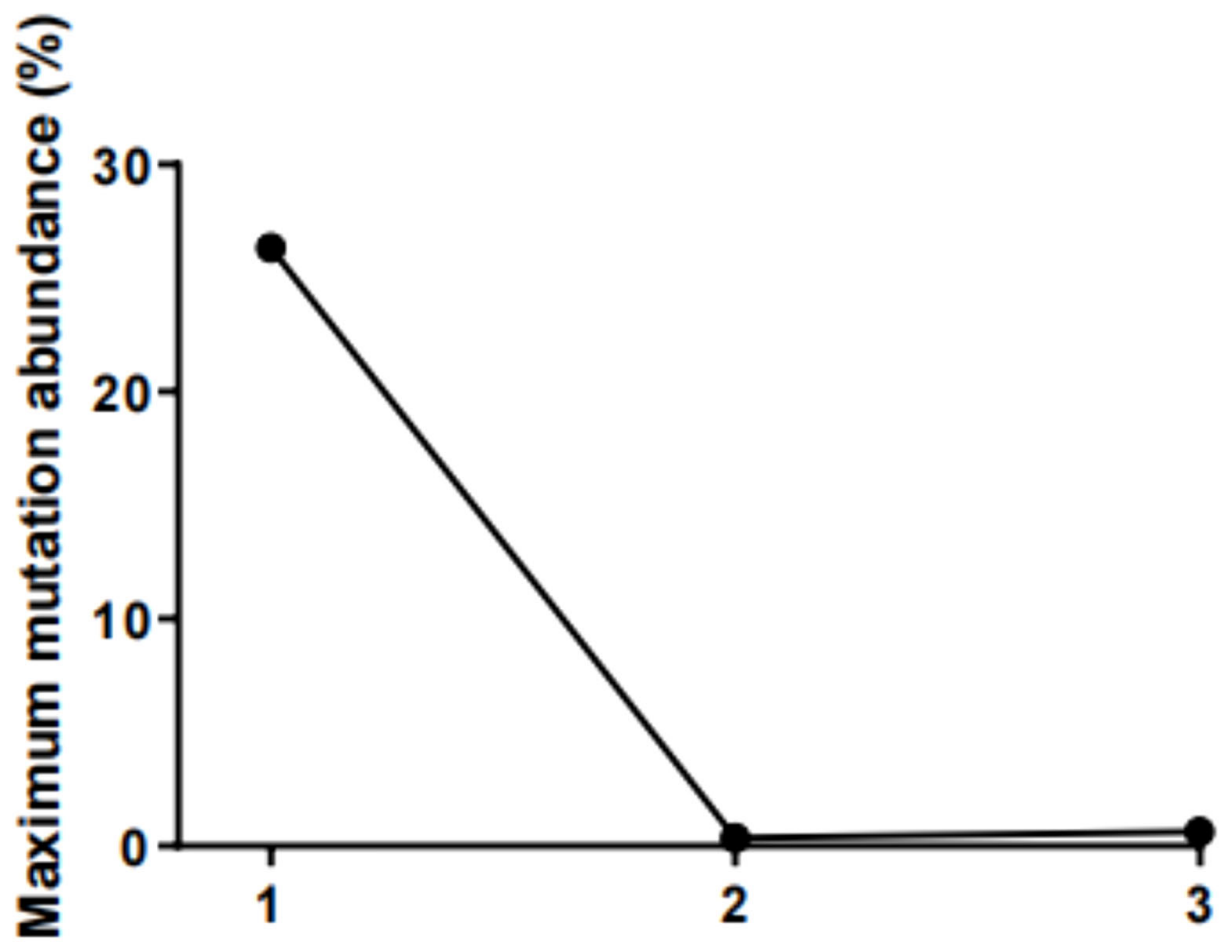
The variation diagram of the maximum mutation abundance of ctDNA.

To investigate serial ctDNA dynamic changes in predicting the tumor response and outcomes in this patient, we collected peripheral blood samples in three periods. At baseline, *CDKN2A* p.A17Gfs*9 (26.34% abundance), *TP53* p.C176* (19.72% abundance), and *KRAS* p.G12V (14.79% abundance) were detected, the TMB value was 1.42 muts/Mb, and the sample presented microsatellite stability status (MSS). These baseline profiles do not suggest that the patient may be a potential immunotherapy beneficiary. Interestingly, two cycles after the initiation of induction therapy, testing result clarified that no baseline gene mutations were detected, except for an extremely low abundance of newly generated *DNMT3A* p.R326C mutation (0.37% abundance). The dynamic changes of ctDNA during treatment with baseline strongly suggested perfect treatment efficacy. Preoperative blood was then collected and tested; in line with the finding during treatment, a low abundance of *MITF* p.R324H mutation (0.62% abundance) was detected, highlighting a process of continuous clinical treatment benefit. The variation diagram of the maximum mutation abundance of ctDNA dynamic changes in three defined time points was shown in **Figure 4**.

## Discussion

Increasing the R0 resection rate and prolonging OS are the main aims of preoperative treatment for initially non-resectable pancreatic cancer. This is a case of induction therapy with significant benefit. Our results suggested that induction anti-PD-1 antibody plus chemotherapy and concurrent radiotherapy may be a potential regimen for patients with LAPC.

PD-1/PD-L1 antibody is currently one of the most used immunotherapies of solid tumors and has shown great advantage in carcinomas such as NSCLC (20) and head and neck squamous cell carcinoma (HNSCC) (21). Microsatellite instability-high (MSI-H) and/or defect mismatch repair (dMMR) gene incidence in pancreatic cancer is less than 1%, which precludes the potential application of PD-1 blockade in pancreatic cancer (22, 23). Furthermore, pancreatic cancer has a denser stroma and a more complex tumor immune microenvironment compared with other solid tumors (24). For the purpose of improving the treatment effect of PD-1 inhibitors, researchers have tried a variety of methods and found that radiotherapy could modulate the tumor environment, thereby improving the antitumor response. The combination pattern has succeeded in NSCLC (25) and esophageal squamous cell carcinoma (26). At present, immune checkpoint inhibitors plus chemotherapy or radiotherapy have also gradually entered the public eye. The combination of chemotherapy and PD-1 antibody showed great advantages in metastatic pancreatic cancer (27), and there was a patient with LAPC who achieved near pCR after the neoadjuvant PD-1 inhibitor plus radiotherapy (28). During the 2021 meeting of the American Association for Cancer Research (AACR), professor Forde concluded the data of the phase III trial Checkmate-816 and suggested that patients in earlier-stage NSCLC who achieved a pCR with neoadjuvant chemotherapy may live longer than those who did not (29). Also, a meta-analysis of randomized phase II/III studies of neoadjuvant therapy for patients with HER-2-positive early breast cancer concluded that patients experienced improved recurrence-free survival (RFS) and OS if they achieved a pCR (30). Thus, it is valid to consider that combined PD-1 inhibitor with chemotherapy and concurrent radiotherapy as induction therapy may be a potential way to improve the resection rate and prolong the survival. This case is the first patient enrolled in our clinical trial who had a pCR after the induction therapy, and we will continue to follow up to record the OS. In addition, the successful surgery of the patient without postoperative complications increased our confidence to continue the clinical trial. We are looking forward to enrolling more subjects and expecting more patients to achieve a pCR to the therapy so we can analyze the role of pCR as a predictor of OS in patients treated with induction therapy.

In this case, we implemented the targeted Next-generation sequencing technology (NGS) using a 539-gene panel to investigate the serial changes of ctDNA status during the treatment. Genomic features at baseline are believed to hold great potential to predict tumor response to immunological induction therapy. However, the Tumor Mutation (TMB) value and MSS status do not suggest that the patient may be a potential immunotherapy beneficiary. Interestingly, ctDNA testing during treatment clarified that no baseline gene mutations were detected, which strongly suggested a perfect treatment efficacy. This is consistent with the changes that reflected in standard imaging and CA19-9 alterations, indicating that serial ctDNA alterations accurately provide a comprehensive outlook of the tumor status and monitor the response to the therapy.

Indeed, serial ctDNA has been proven to predict and monitor the effect of neoadjuvant chemoradiotherapy in patients with rectal cancers; ctDNA becomes a real-time monitoring indicator that can accurately reflect the tumor burden. The median variant allelic frequency (VAF) of baseline ctDNA is a strong independent predictor of survival outcome (31). In addition, ctDNA can also serve as a potential biomarker of the response to immunotherapy in advanced gastric cancers, and ctDNA presented a potential role in predicting Immune-Related Adverse Events (irAEs) (32).

## Conclusion

In conclusion, we provide a reference for the use of induction PD-1 blockade plus chemoradiotherapy in patients with potentially resectable pancreatic cancer. This is a typical case that proves the prognostic value of ctDNA in pancreatic cancer. However, there are some limitations, and more randomized clinical trials are needed to verify the safety and efficacy of the induction therapy mode in LAPC and BRPC. Furthermore, evidence of the ctDNA change in a single case is not very valuable; these differences should be further validated in a larger cohort.

## Data Availability Statement

The original contributions presented in the study are included in the article/supplementary material. Further inquiries can be directed to the corresponding authors.

## Ethics Statement

The studies involving human participants were reviewed and approved by the Medical Ethics Committee of Drum Tower Hospital Affiliated to Nanjing University Medical School. The patients/participants provided their written informed consent to participate in this study. Written informed consent was obtained from the individual(s) for the publication of any potentially identifiable images or data included in this article.

## Author Contributions

CL, YZ, and HC have contributed equally to this work. BL, YQ, and JD designed the clinical trial. CL, YZ, and HC composed the article. MT, JH, and AL provided the imaging report, and JC and QL provided the pathology report. WK, LW and XgQ revised the article. LZ, XnQ, FM and DC did the work of the acquisition, analysis, and interpretation of the data. All authors have agreed to the final version of the article.

## Funding

National Natural Science Foundation of China (82072926); National Key Research and Development Program of China (2020YFA0713804); Special Fund of Health Science and Technology Development of Nanjing (YKK20080)

## Conflict of Interest

Author DC was employed by The State Key Laboratory of Translational Medicine and Innovative Drug Development, Medical Department, Jiangsu Simcere Diagnostics Co., Ltd., Nanjing, China.

The remaining authors declare that the research was conducted in the absence of any commercial or financial relationships that could be construed as a potential conflict of interest.

## Publisher’s Note

All claims expressed in this article are solely those of the authors and do not necessarily represent those of their affiliated organizations, or those of the publisher, the editors and the reviewers. Any product that may be evaluated in this article, or claim that may be made by its manufacturer, is not guaranteed or endorsed by the publisher.
